# Supplementary material for: Oleic Acid Exhibits Anti-Proliferative and Anti-Invasive Activities via the PTEN/AKT/mTOR Pathway in Endometrial Cancer
Source: Cancers (Basel). 2023 Nov 14;15(22):5407. doi: 10.3390/cancers15225407 (PMC10670880; doi:10.3390/cancers15225407)

Figure S1: Effect of OA on the expression of proteins related to cellular stress, autophagy, and cell apoptosis in EC.

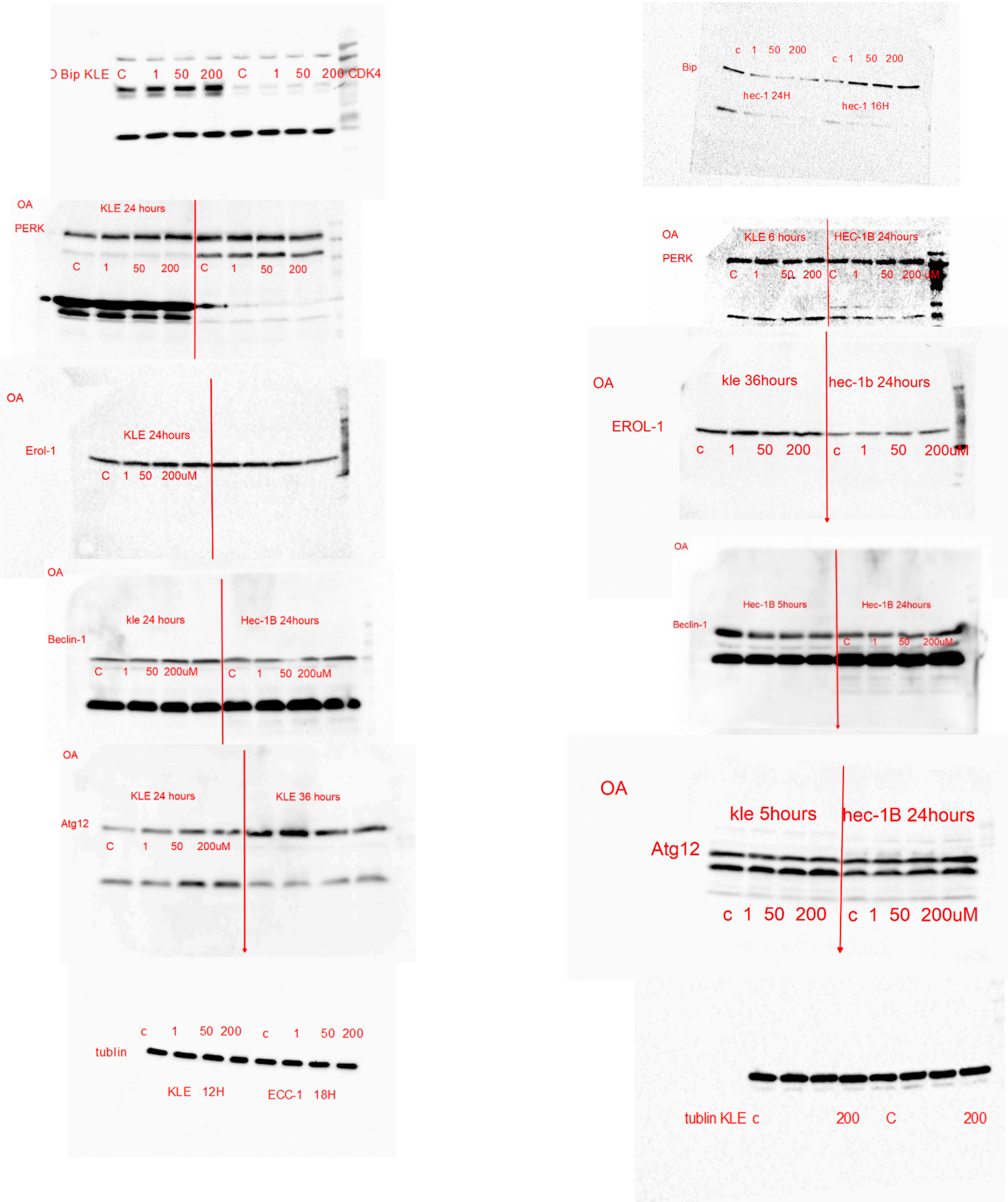

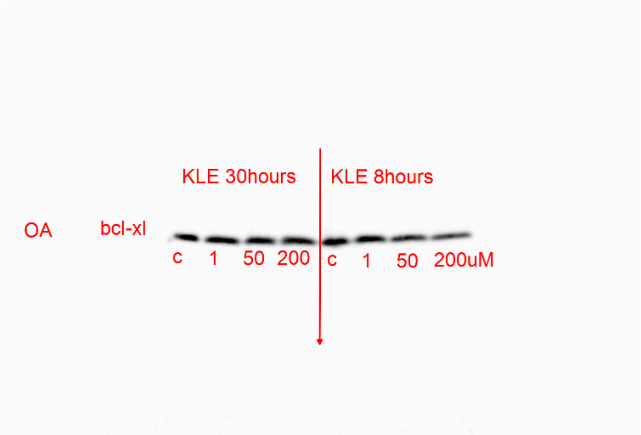
KLE 30hours
KLE 8hours
OA
bcl-xl
c 1 50 200 c 1 50 200uM

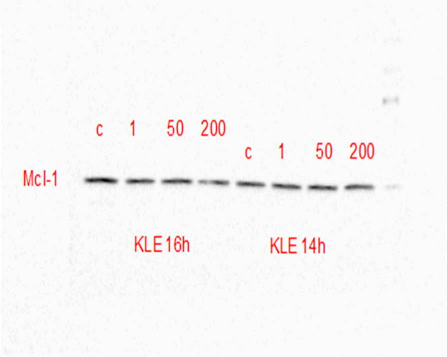
c 1 50 200
c 1 50 200
Mcl-1
KLE16h
KLE14h

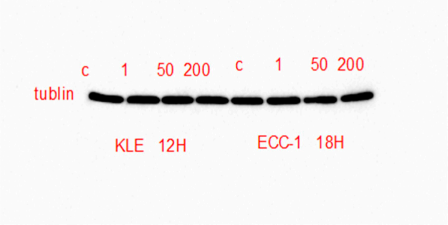
c 1 50 200 c 1 50 200
tublin
KLE 12H
ECC-1 18H

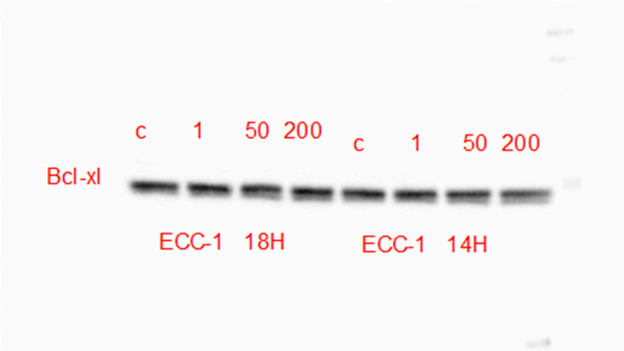
c 1 50 200 c 1 50 200
Bcl-xl
ECC-1 18H
ECC-1 14H

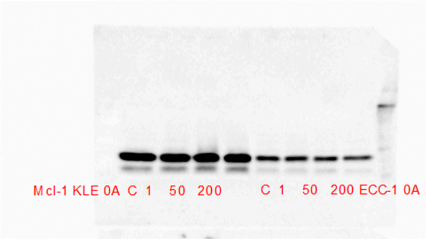
Mcl-1 KLE 0A C 1 50 200 C 1 50 200 ECC-1 0A

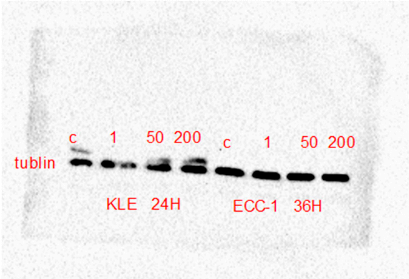
c 1 50 200 c 1 50 200
tublin
KLE 24H
ECC-1 36H

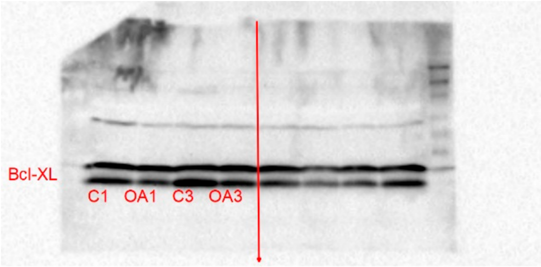
Bcl-XL
C1 OA1 C3 OA3

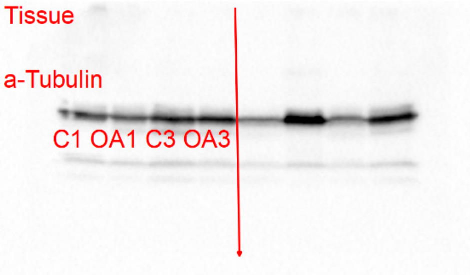
Tissue
a-Tubulin
C1 OA1 C3 OA3

Figure S2: OA decreased the expression of proteins related to cell cycle in EC cells.

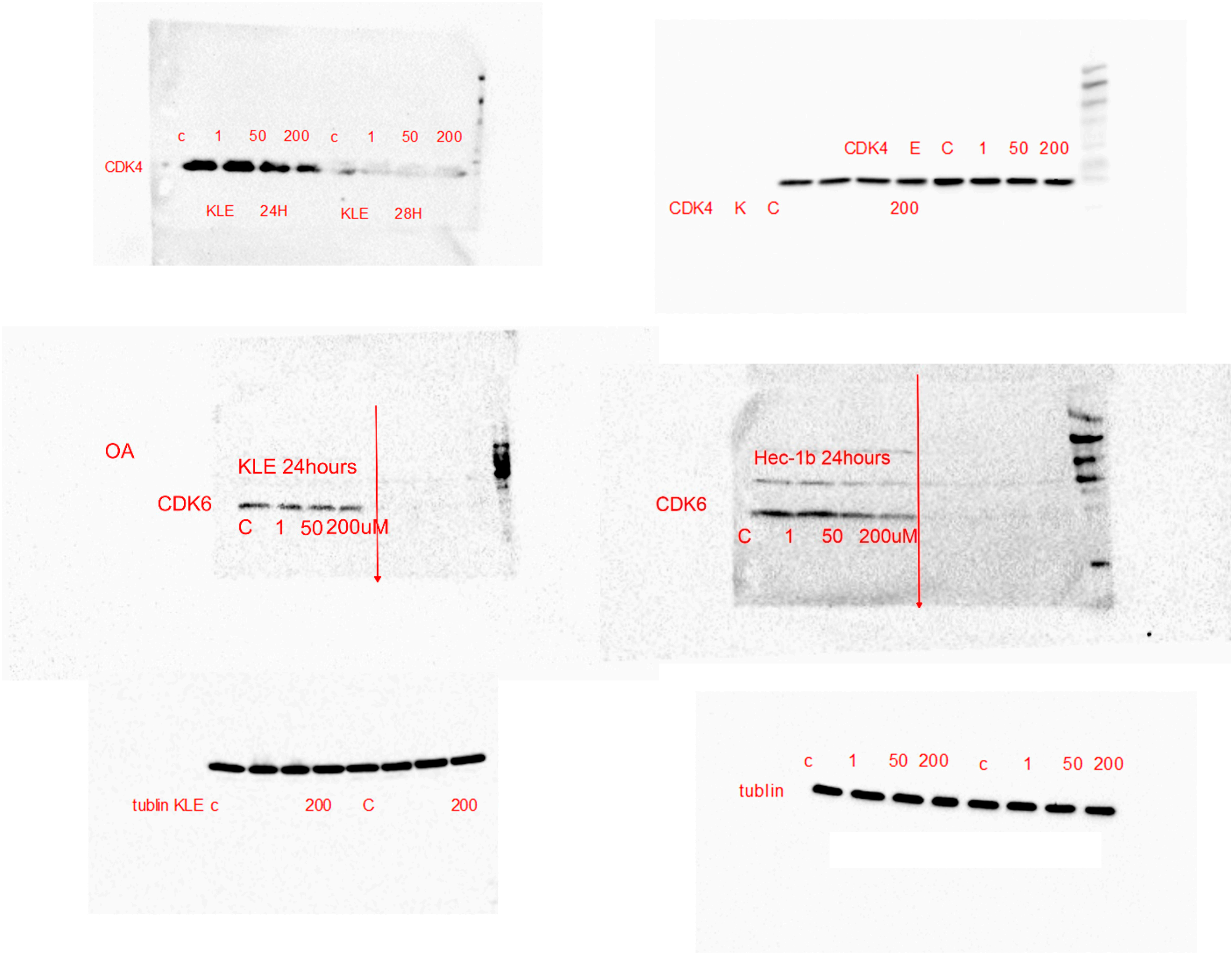

Figure S3: Effect of OA on the expression of proteins related to EMT in EC cells.

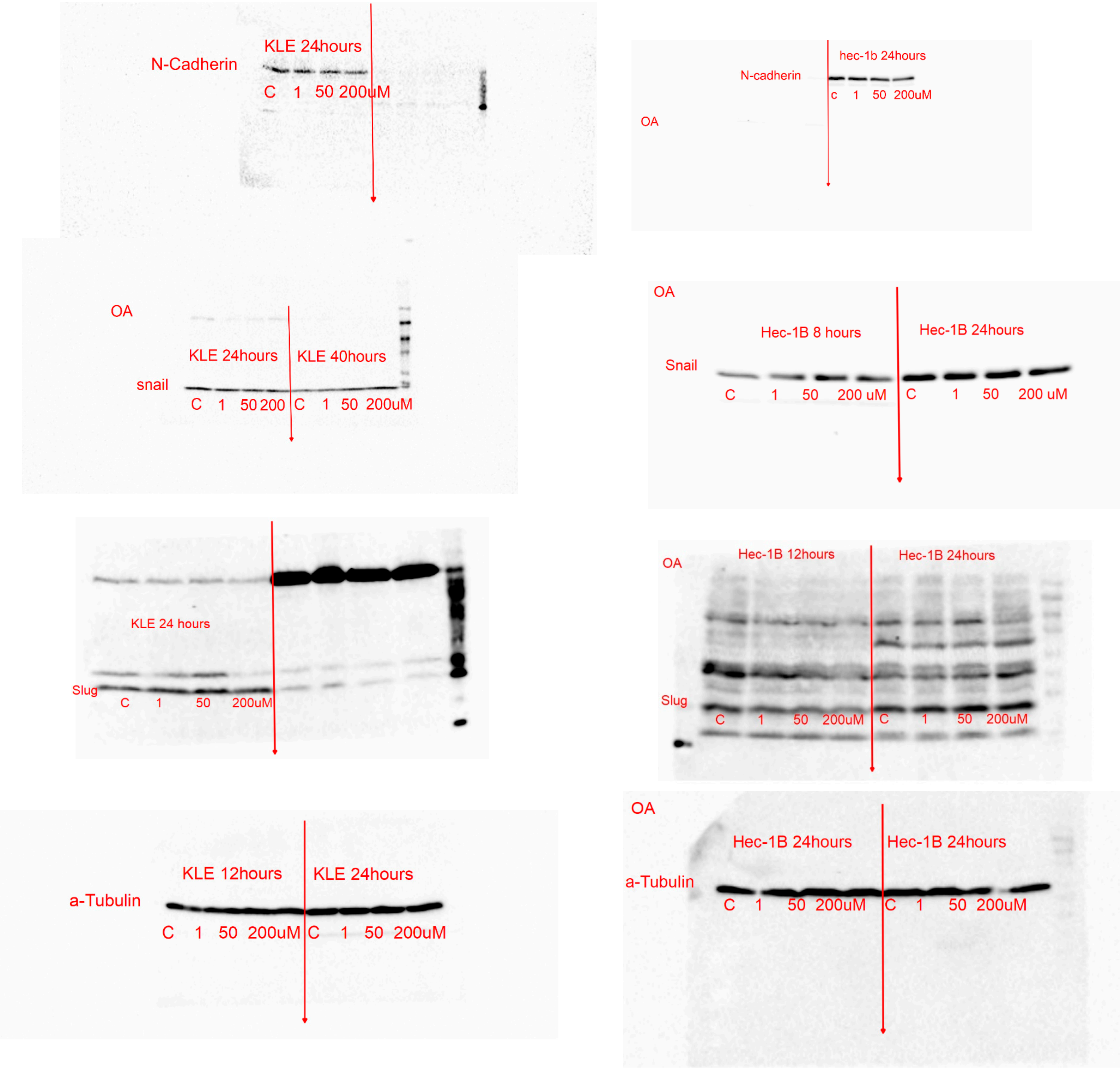

Figure S4: Effect of OA on the expression of proteins related to fatty acid metabolism in EC cells.

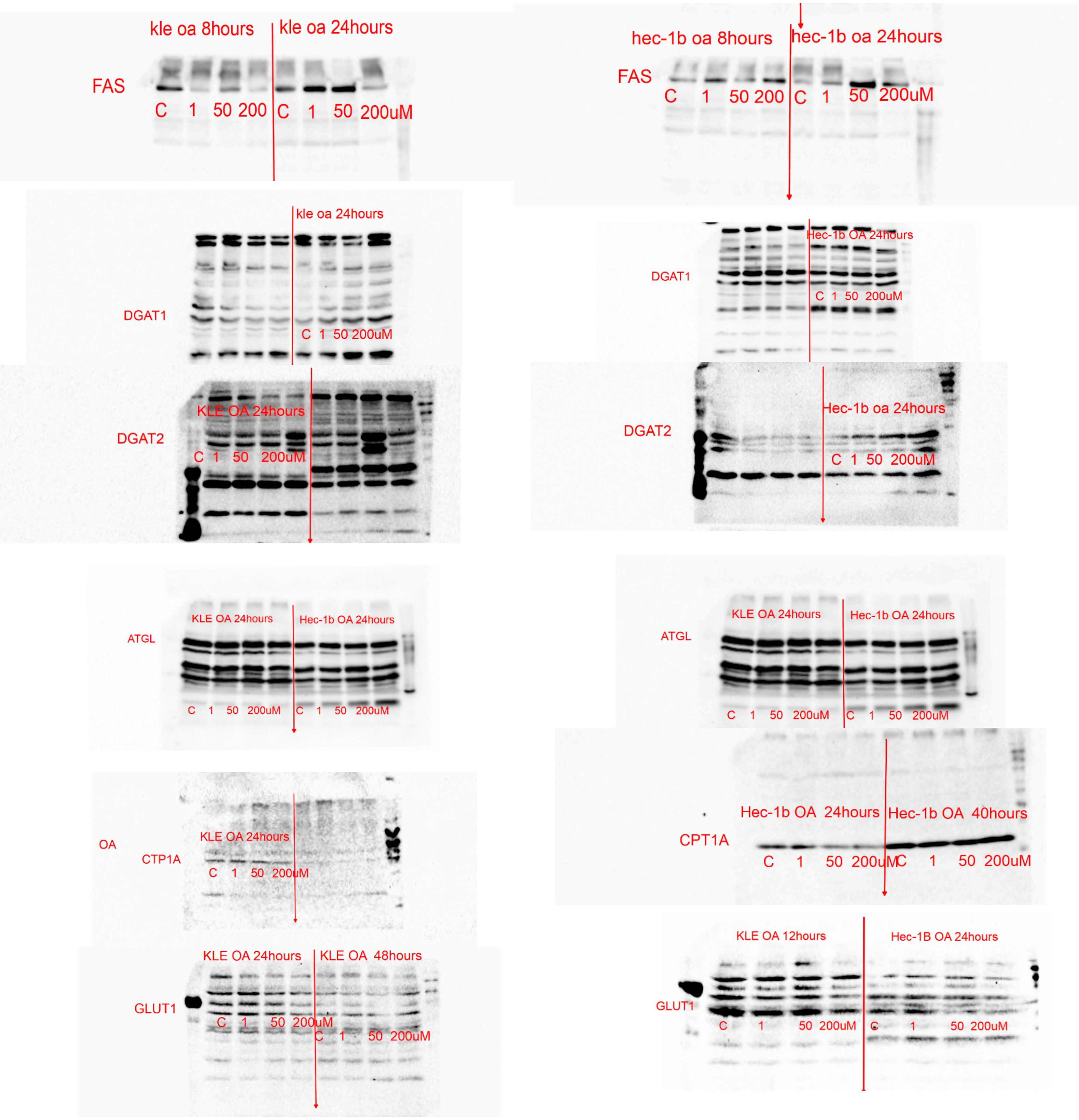

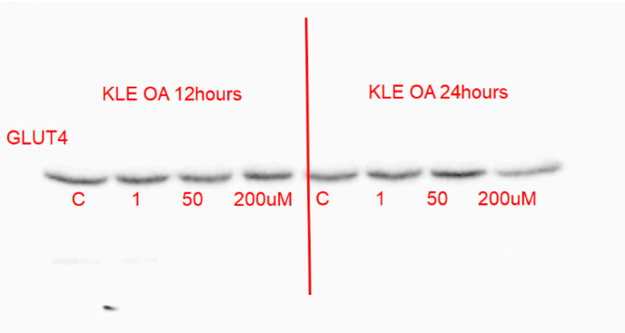
KLE OA 12hours
KLE OA 24hours
GLUT4
C 1 50 200uM C 1 50 200uM

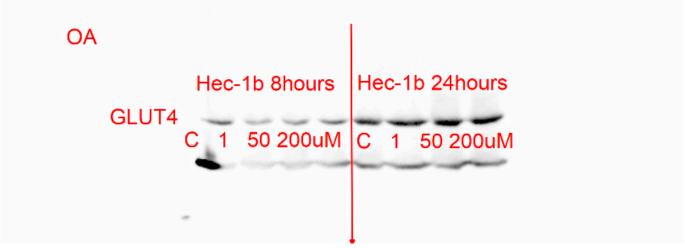
OA
Hec-1b 8hours
Hec-1b 24hours
GLUT4
C 1 50 200uM C 1 50 200uM

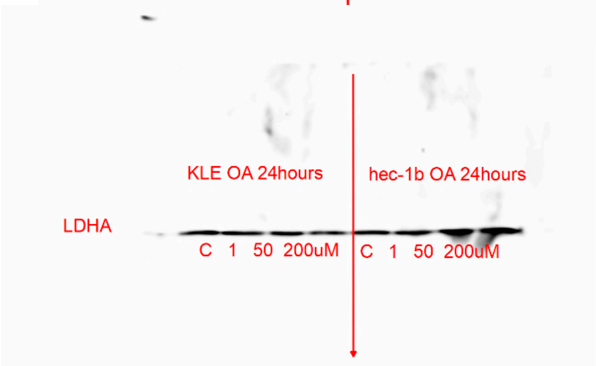
KLE OA 24hours
hec-1b OA 24hours
LDHA
C 1 50 200uM C 1 50 200uM

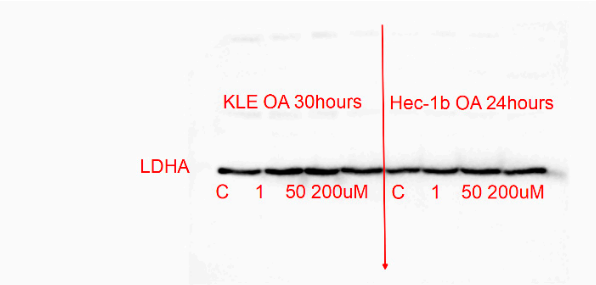
KLE OA 30hours
Hec-1b OA 24hours
LDHA
C 1 50 200uM C 1 50 200uM

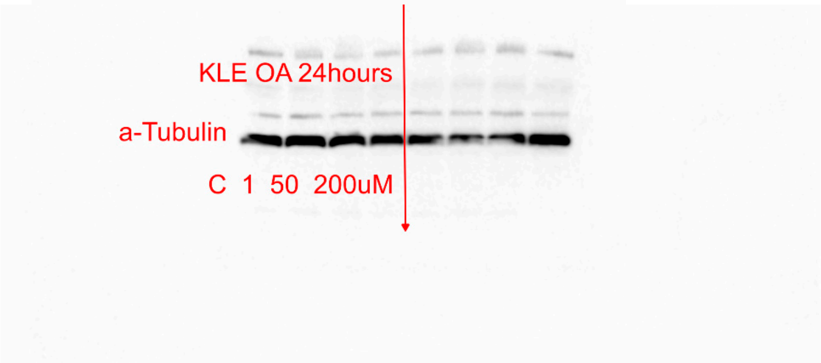
KLE OA 24hours
a-Tubulin
C 1 50 200uM

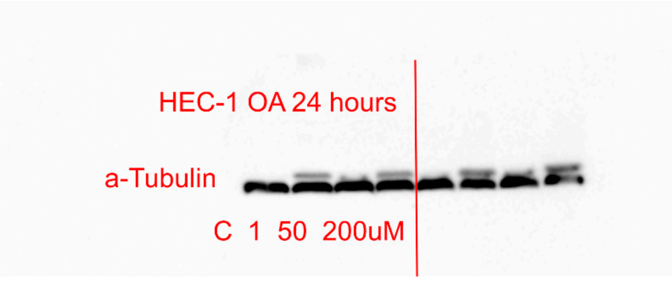
HEC-1 OA 24 hours
a-Tubulin
C 1 50 200uM

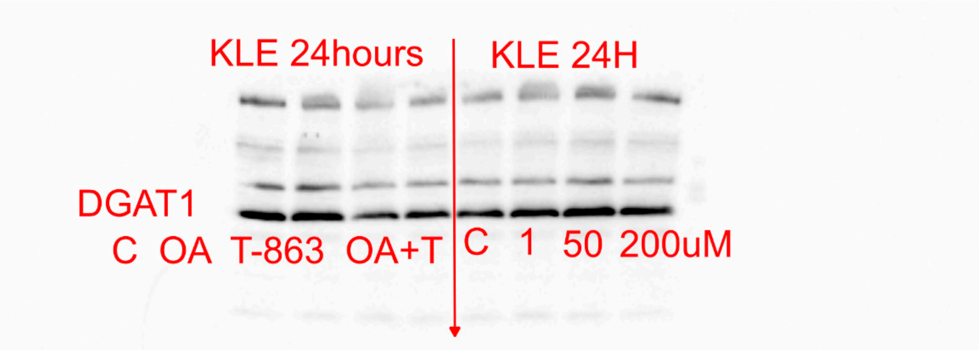
KLE 24hours
KLE 24H
DGAT1
C OA T-863 OA+T
C 1 50 200uM

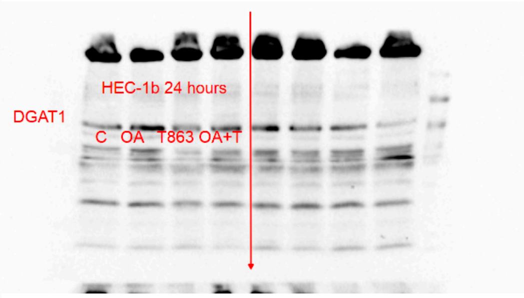
HEC-1b 24 hours
DGAT1
C OA T863 OA+T

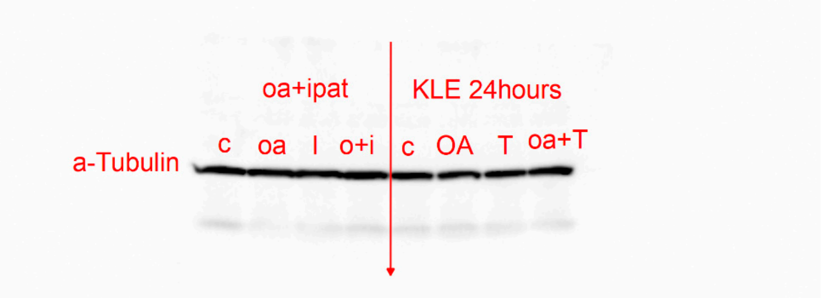
oa+ipat
KLE 24hours
a-Tubulin
c oa I o+i
c OA T oa+T

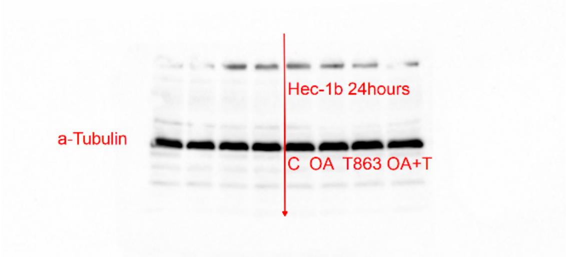
Hec-1b 24hours
a-Tubulin
C OA T863 OA+T

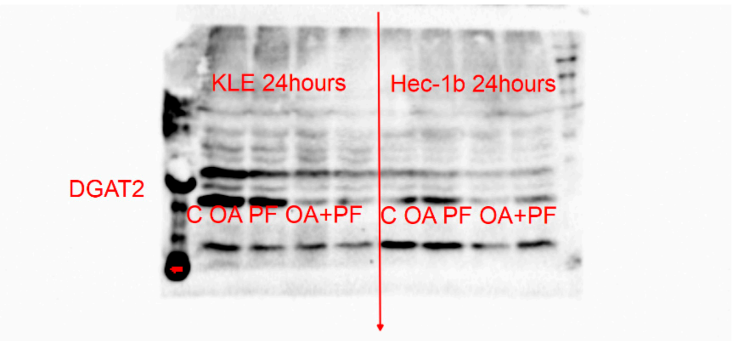
KLE 24hours
Hec-1b 24hours
DGAT2
C OA PF OA+PF
C OA PF OA+PF

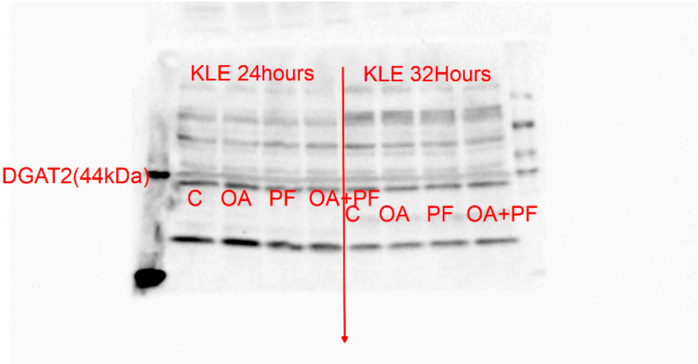
KLE 24hours
KLE 32Hours
DGAT2(44kDa)
C OA PF OA+PF
C OA PF OA+PF

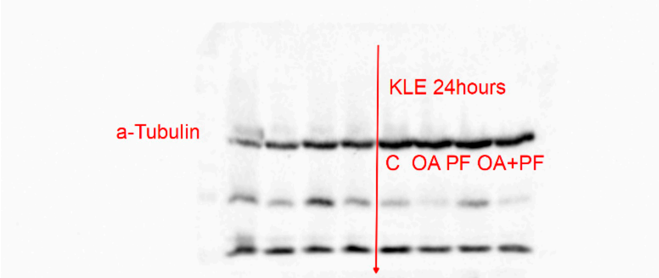
KLE 24hours
a-Tubulin
C OA PF OA+PF

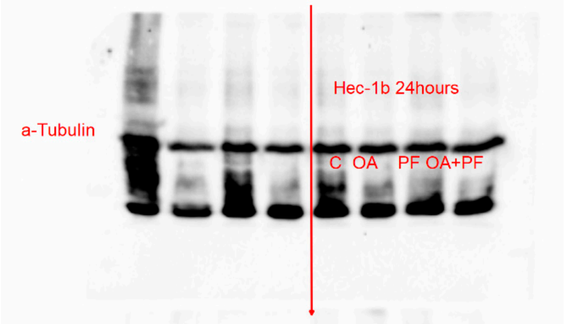
Hec-1b 24hours
a-Tubulin
C OA PF OA+PF

Figure S5: Effect of OA on the expression of proteins related to PTEN/AKT/mTOR and MAPK pathways in EC cells.

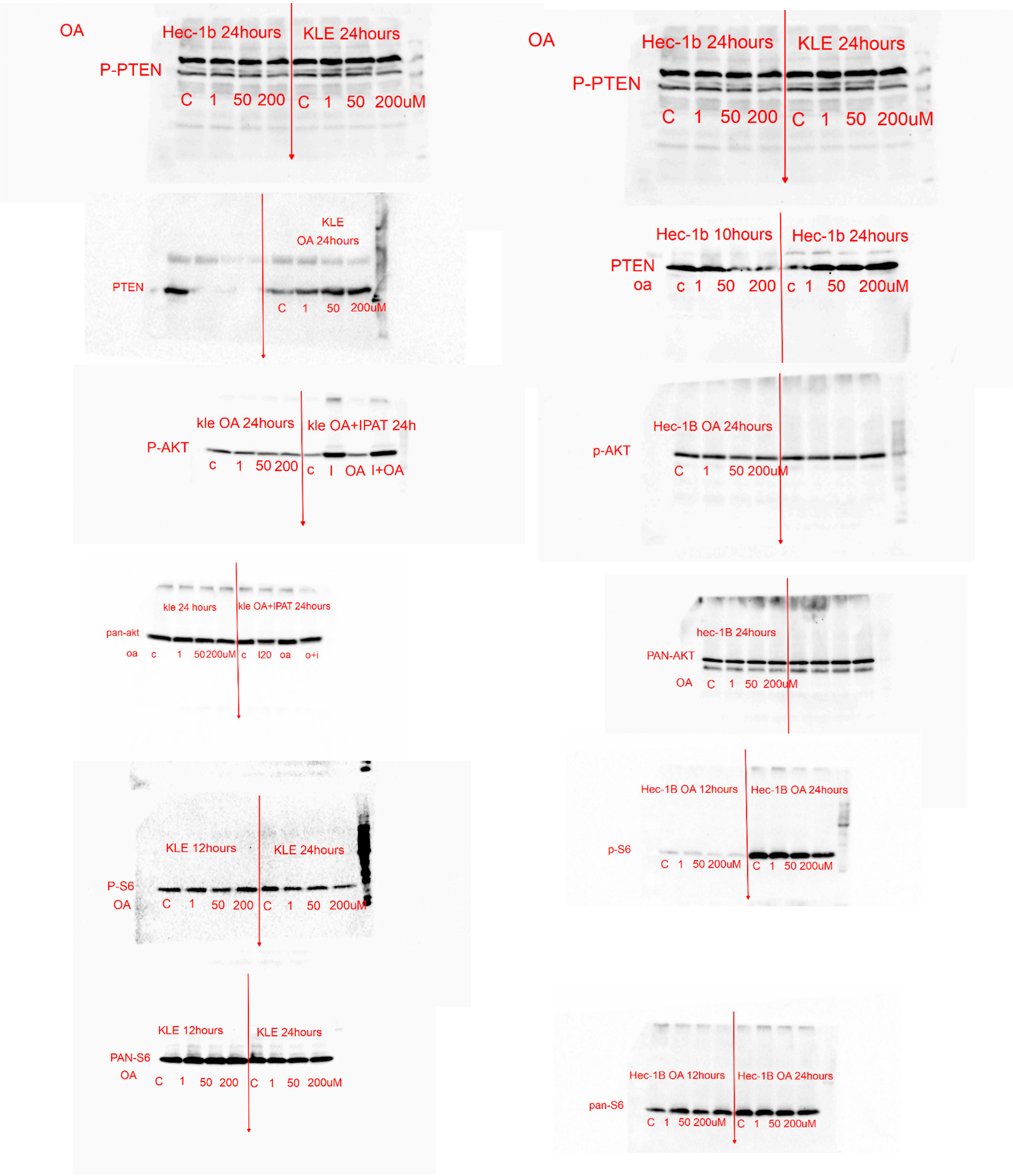

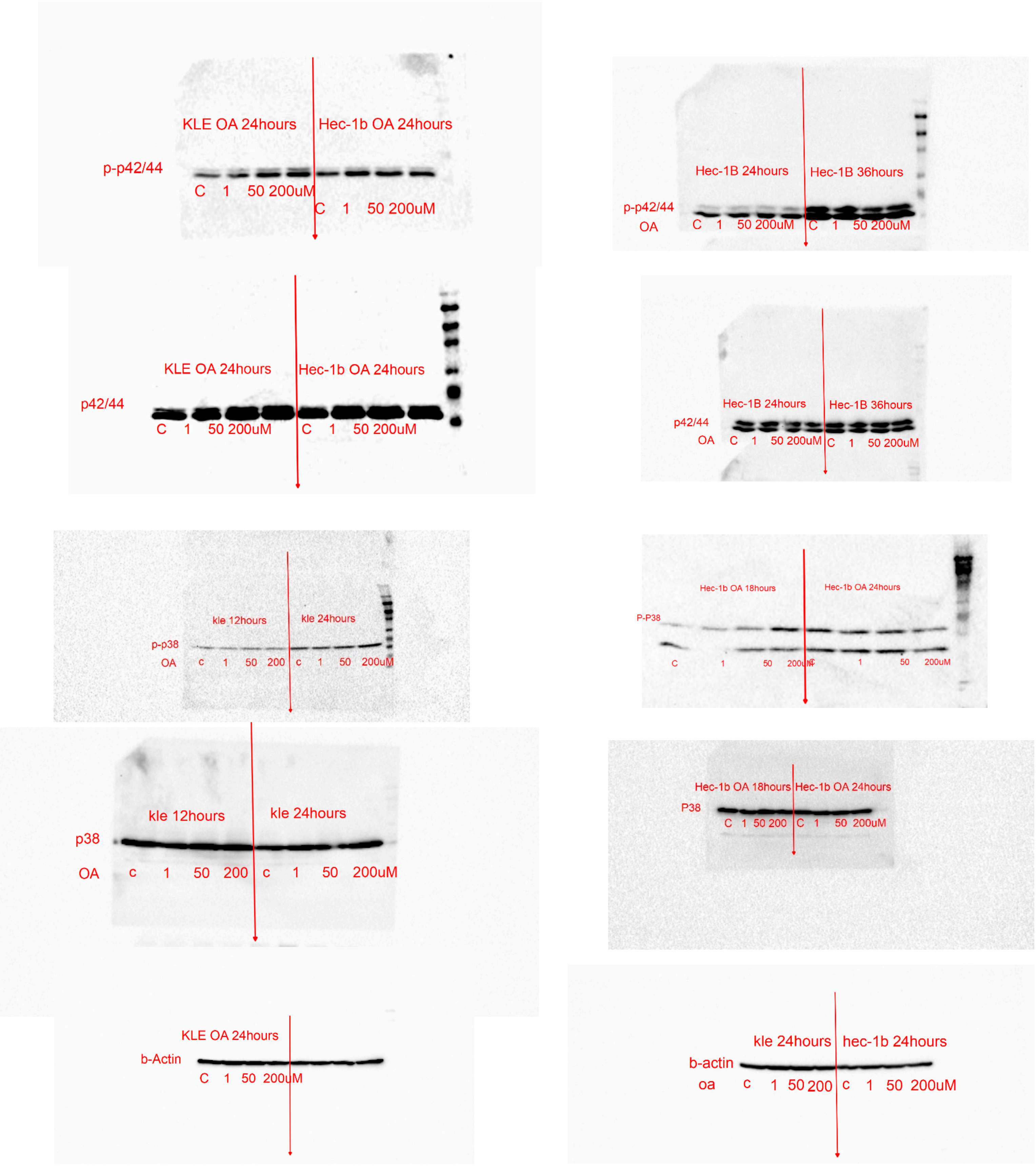
KLE OA 24hours
Hec-1b OA 24hours
p-p42/44
C 1 50 200uM
C 1 50 200uM
Hec-1B 24hours
Hec-1B 36hours
p-p42/44
OA
C 1 50 200uM
C 1 50 200uM
KLE OA 24hours
Hec-1b OA 24hours
p42/44
C 1 50 200uM
C 1 50 200uM
Hec-1B 24hours
Hec-1B 36hours
p42/44
OA
C 1 50 200uM
C 1 50 200uM
kle 12hours
kle 24hours
p-p38
OA
c 1 50 200
c 1 50 200uM
Hec-1b OA 18hours
Hec-1b OA 24hours
P-P38
C 1 50 200uM
C 1 50 200uM
kle 12hours
kle 24hours
p38
OA
c 1 50 200
c 1 50 200uM
Hec-1b OA 18hours
Hec-1b OA 24hours
P38
C 1 50 200
C 1 50 200uM
KLE OA 24hours
b-Actin
C 1 50 200uM
kle 24hours
hec-1b 24hours
b-actin
oa
c 1 50 200
c 1 50 200uM

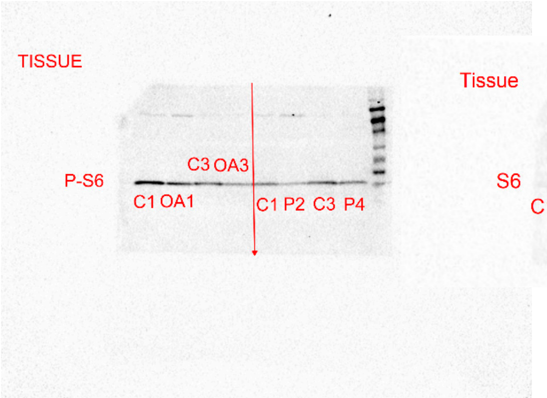
TISSUE
P-S6
C3 OA3
C1 OA1
C1 P2 C3 P4

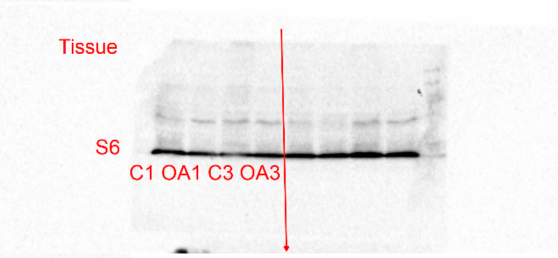
Tissue
S6
C1 OA1 C3 OA3

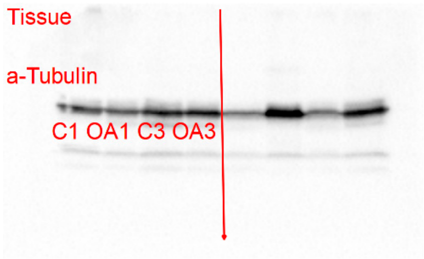
Tissue
a-Tubulin
C1 OA1 C3 OA3

Figure S6: PTEN knockdown led to the change of protein expression in HEC-1B cells and partially reversed the inhibitory effects of OA on the related protein expression.

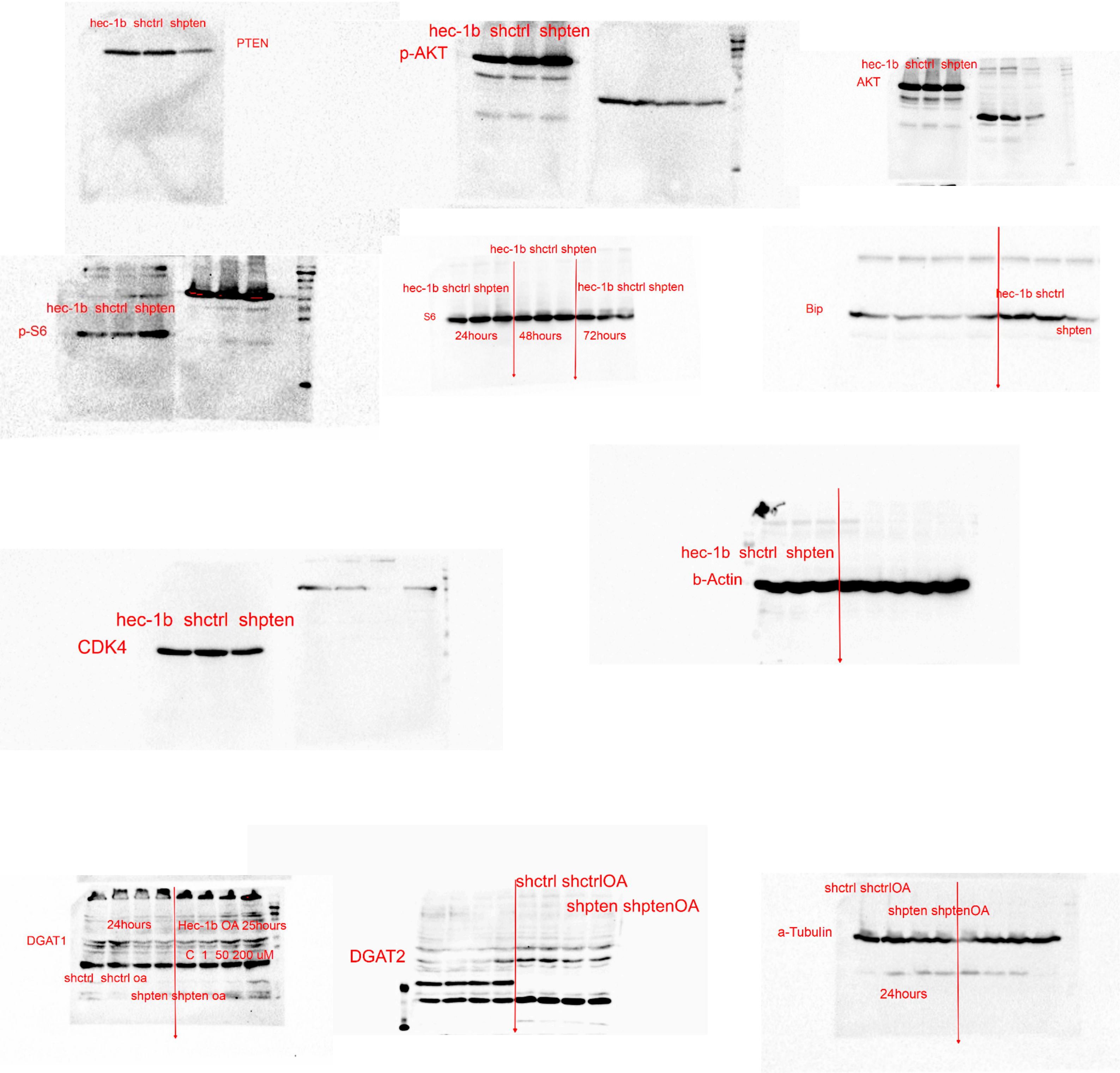

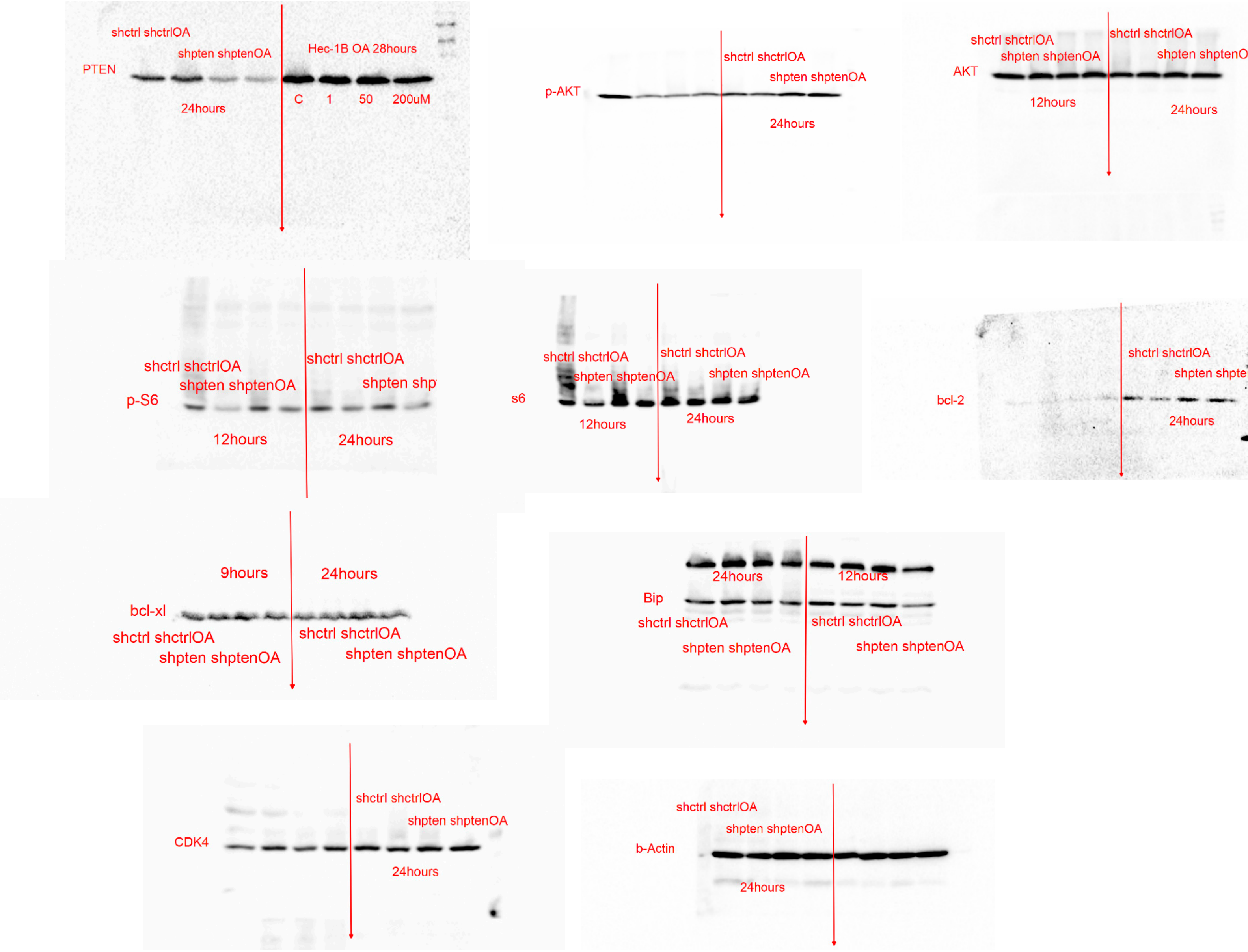
shctrl shctrlOA
shpten shptenOA
PTEN
Hec-1B OA 28hours
C 1 50 200uM
24hours
p-AKT
shctrl shctrlOA
shpten shptenOA
24hours
AKT
shctrl shctrlOA
shpten shptenOA
12hours
24hours
p-S6
shctrl shctrlOA
shpten shptenOA
shpten shp
12hours
24hours
s6
shctrl shctrlOA
shpten shptenOA
12hours
24hours
bcl-2
shctrl shctrlOA
24hours
bcl-xl
9hours
24hours
shctrl shctrlOA
shpten shptenOA
Bip
24hours
12hours
shctrl shctrlOA
shpten shptenOA
CDK4
shctrl shctrlOA
shpten shptenOA
24hours
b-Actin
shctrl shctrlOA
shpten shptenOA
24hours

Figure S7: The combination of IPAT and OA showed the greatest effects on the expression of related protein than either agent alone.

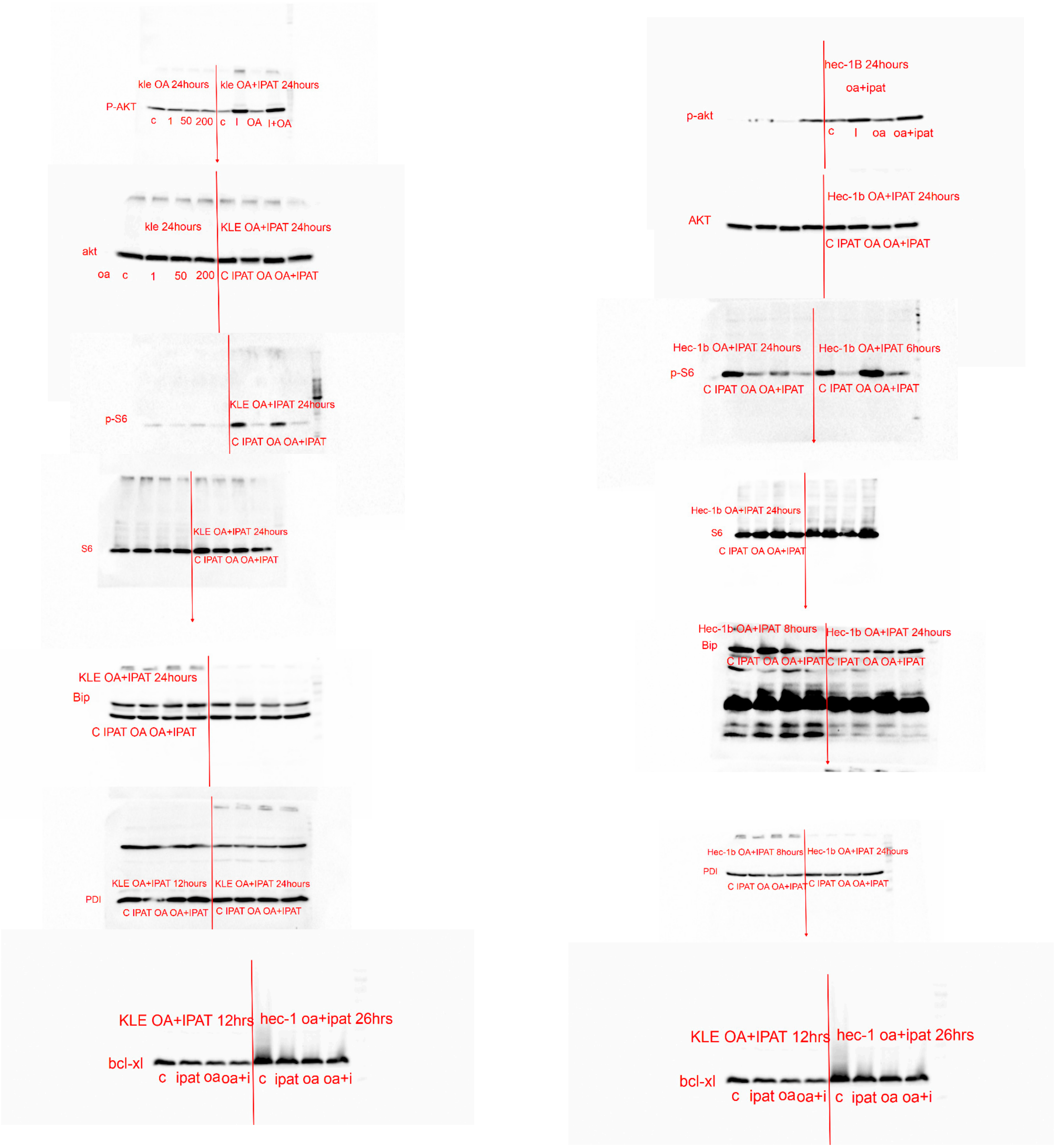

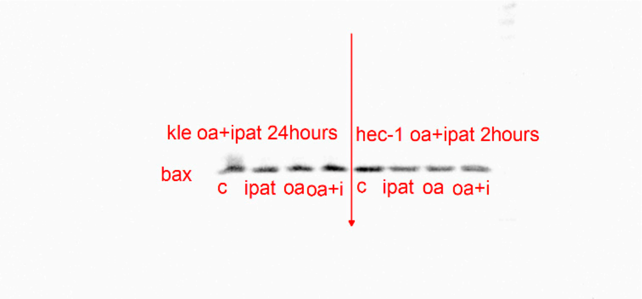
kle oa+ipat 24hours
hec-1 oa+ipat 2hours
bax
c ipat oa oa+i
c ipat oa oa+i

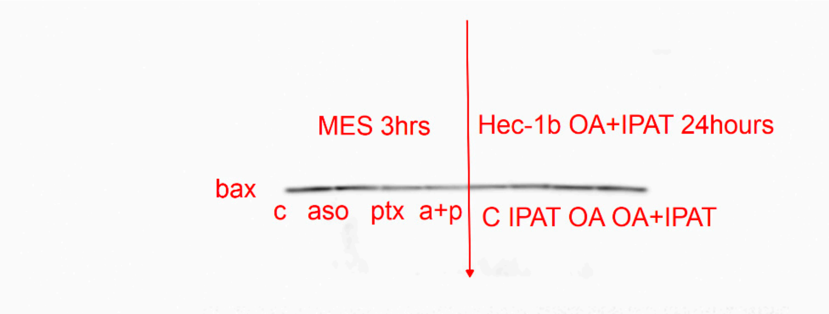
MES 3hrs
Hec-1b OA+IPAT 24hours
bax
c aso ptx a+p
C IPAT OA OA+IPAT

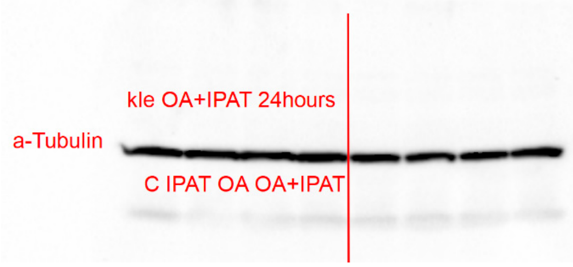
kle OA+IPAT 24hours
a-Tubulin
C IPAT OA OA+IPAT

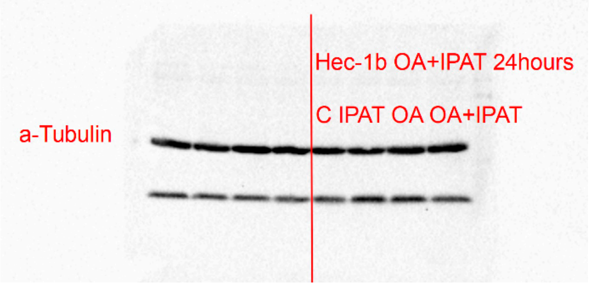
Hec-1b OA+IPAT 24hours
C IPAT OA OA+IPAT
a-Tubulin

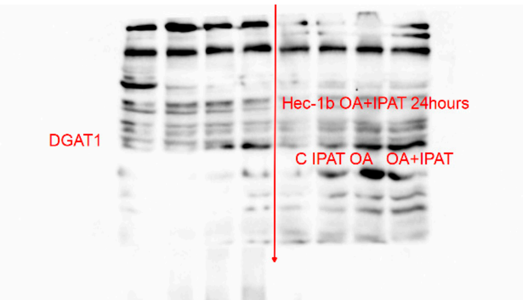
Hec-1b OA+IPAT 24hours
DGAT1
C IPAT OA OA+IPAT

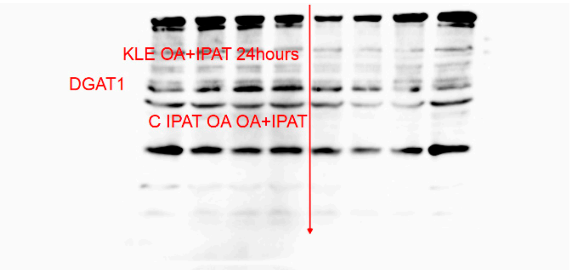
KLE OA+IPAT 24hours
DGAT1
C IPAT OA OA+IPAT

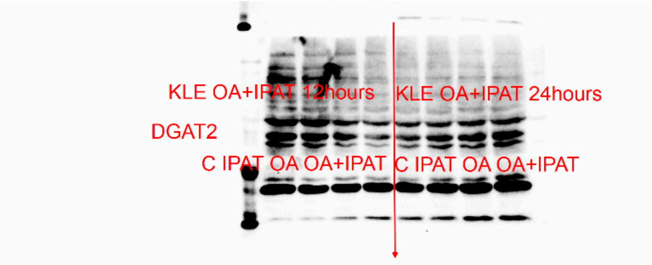
KLE OA+IPAT 12hours
KLE OA+IPAT 24hours
DGAT2
C IPAT OA OA+IPAT
C IPAT OA OA+IPAT

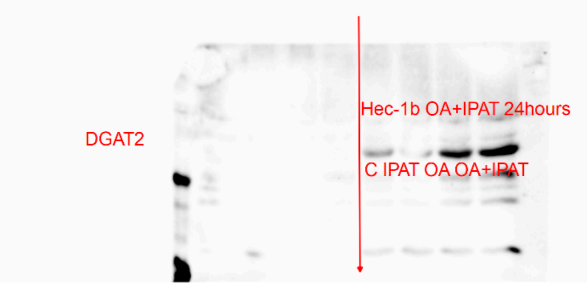
Hec-1b OA+IPAT 24hours
DGAT2
C IPAT OA OA+IPAT

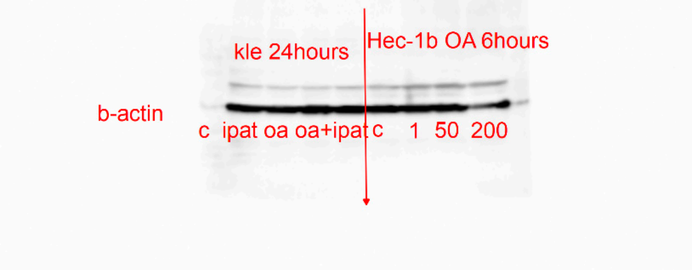
kle 24hours
Hec-1b OA 6hours
b-actin
c ipat oa oa+ipat
c 1 50 200

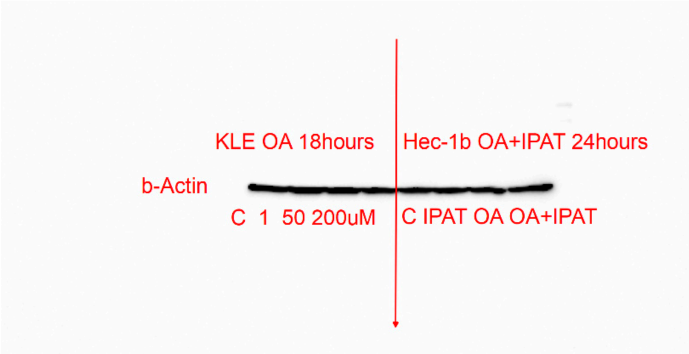
KLE OA 18hours
Hec-1b OA+IPAT 24hours
b-Actin
C 1 50 200uM
C IPAT OA OA+IPAT

Figure S8: OA inhibited the expression of proteins related to EMT via PTEN/AKT/S6 pathways in EC cells.

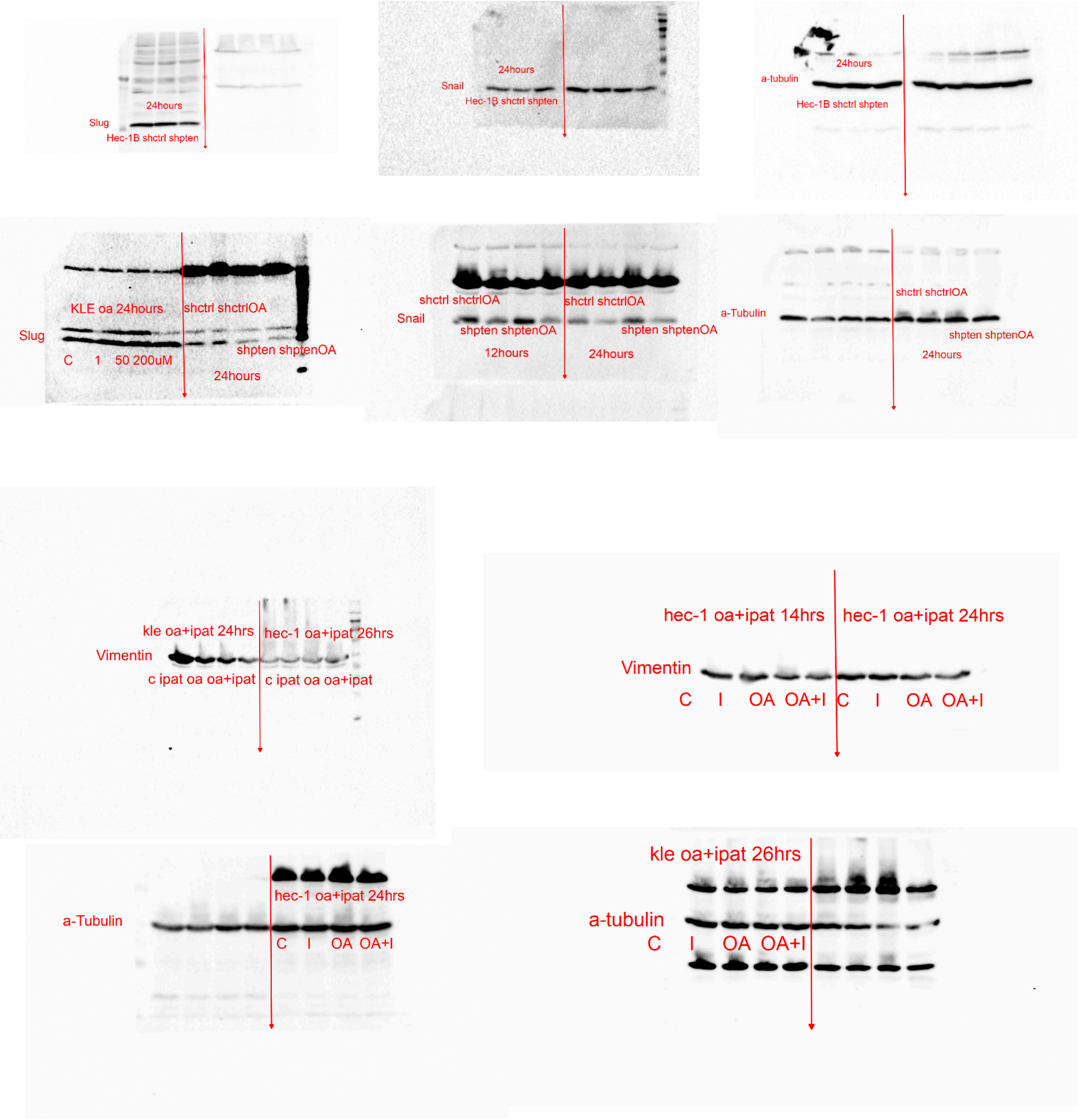

Supplement: Supplementary file 1 [file cancers-15-05407-s001.zip › cancers-2681524-supplementary.pdf]
